# Understanding the Pathophysiology of Alzheimer's Disease and Mild Cognitive Impairment: A Mini Review on fMRI and ERP Studies

**DOI:** 10.1155/2012/719056

**Published:** 2011-07-07

**Authors:** Takao Yamasaki, Hiroyuki Muranaka, Yumiko Kaseda, Yasuyo Mimori, Shozo Tobimatsu

**Affiliations:** ^1^Department of Clinical Neurophysiology, Neurological Institute, Graduate School of Medical Sciences, Kyushu University, 3-1-1 Maidashi, Higashi-ku, Fukuoka 812-8582, Japan; ^2^Department of Neurology, Minkodo Minohara Hospital, 3553 Kanaide, Sasaguri-machi, Kasuya-gun, Fukuoka 811-2402, Japan; ^3^Department of Radiology, Hiroshima City General Rehabilitation Center, 1-39-1 Tomo-minami, Asaminami-ku, Hiroshima 731-3168, Japan; ^4^Department of Neurology, Hiroshima City General Rehabilitation Center, 1-39-1 Tomo-minami, Asaminami-ku, Hiroshima 731-3168, Japan; ^5^Department of Physical Therapy, Faculty of Health Sciences, Hiroshima International University, 555-36 Gakuenndai, Kurose, Higashihiroshima, Hiroshima 739-2695, Japan

## Abstract

The prevalence of Alzheimer's disease (AD) is predicted to increase rapidly in the coming decade, highlighting the importance of early detection and intervention in patients with AD and mild cognitive impairment (MCI). Recently, remarkable advances have been made in the application of neuroimaging techniques in investigations of AD and MCI. Among the various neuroimaging techniques, functional magnetic resonance imaging (fMRI) has many potential advantages, noninvasively detecting alterations in brain function that may be present very early in the course of AD and MCI. In this paper, we first review task-related and resting-state fMRI studies on AD and MCI. We then present our recent fMRI studies with additional event-related potential (ERP) experiments during a motion perception task in MCI. Our results indicate that fMRI, especially when combined with ERP recording, can be useful for detecting spatiotemporal functional changes in AD and MCI patients.

## 1. Introduction

Dementia is one of the most serious conditions associated with longevity, and represents a pressing public health problem. Alzheimer's disease (AD) is the most common form of dementia, affecting millions of people around the world. AD is a progressive neurodegenerative disorder, resulting in a gradual, irreversible loss of memory and cognitive function [1]. Recently recognized as the prodromal stage of AD, mild cognitive impairment (MCI) represents a transitional period between normal aging and AD [2, 3]. MCI pathology can reveal the early stages of AD, including neuritic plaques, neurofibrillary tangles, and loss of basal forebrain cholinergic neurons [4]. As a subtype of MCI, amnestic MCI constitutes a syndrome presenting with cognitive decline that is more pronounced than expected for the individual's age and educational level, but does not fulfill the criteria for AD. Patients with amnestic MCI have a high risk of AD progression, with a 10–15% yearly transition rate [5]. In addition, large numbers of novel compounds, which have the potential to modify the course of AD and slow its progression, are currently under development. However, there is currently no cure for the disease. For this reason, there is an urgent need for biomarkers to detect MCI.

In the past two decades, several functional imaging techniques have been used to investigate changes in brain function in patients with AD and MCI. Functional magnetic resonance imaging (fMRI), positron emission tomography (PET) and single-photon emission computed tomography (SPECT) are effective methods. Among these techniques, fMRI has a number of potential advantages for examining patients with AD and MCI, since it is noninvasive, does not require the injection of contrast agent, and has much higher spatial resolution than PET or SPECT. Furthermore, fMRI can be conducted many times over the course of a longitudinal study, and thus lends itself as an appropriate measure in clinical drug trials. Therefore, fMRI is likely to be particularly useful for detecting alterations in brain function that may be present very early in the course of AD and MCI. The measurement of event-related potentials (ERPs) represents a useful objective tool, and has been employed extensively in studying the physiology and pathophysiology of human brain function [6]. ERPs are characterized by extremely high temporal resolution, and can allow non-invasive assessment of synaptic dysfunction in the human brain [7]. Thus, the use of fMRI and ERPs together can provide a powerful tool for examining functional brain abnormalities in AD and MCI. 

In this article, we first review previous fMRI studies on AD and MCI. We then present the findings of our recent fMRI studies with additional ERP recording experiments during a motion perception task in MCI patients.

## 2. fMRI Findings in AD and MCI

fMRI has been used to investigate abnormalities in patterns of regional brain activation during a variety of cognitive tasks in patients with AD and MCI. In particular, difficulty in the formation and retention of new episodic memories is typically the earliest and most salient clinical symptom of AD [8–10]. Even in the early stages of the disease, many patients with AD also exhibit visuospatial deficits [11–13]. A large number of fMRI studies have examined memory and visual tasks relative to other types of cognitive task. Furthermore, the recently developed method of measuring resting-state (rs-)fMRI has been used to examine resting brain function in AD and MCI patients [14–16]. Below we review studies using task-related (i.e., during memory and visual tasks) fMRI, as well as rs-fMRI studies in patients with AD and MCI.

### 2.1. Memory Networks

The term “memory” represents a simplified summarization of a wide-ranging set of different associated functions, including short-term, long-term, procedural, declarative, semantic, and episodic memory. Memory can be subdivided into functions related either to the encoding or retrieval of information. The term “declarative memory” refers to the aspect of human memory involved in the storage of facts and experiences, which can be explicitly discussed or declared by the individual. Declarative memory is subdivided into semantic memory (noncontext specific fact, word and object memory) and episodic memory (memory of events, including time, place, and associated emotions). In patients with AD, episodic memory is among the earliest affected functions [8–10]. AD typically results in a deficit in the establishment of new episodic memories whereas events dating from more remote periods in the past are better preserved. In the later stages of AD, most other memory domains are also affected. As such, memory impairment is also a fundamental criterion of the diagnosis of AD [17]. 

The functional neuroanatomy of memory is currently widely believed that many memory functions are tightly linked to distributed regions in the brain. In particular, a neural system in the medial temporal lobe (MTL), including the hippocampal region, and the adjacent perirhinal, entorhinal and parahippocampal cortices, is thought to be involved in encoding and retrieving episodic memory [18–20]. In addition, the prefrontal cortical regions, temporoparietal junction, posterior cingulate cortex, and the cerebellum have been identified as contributing to episodic memory [20, 21]. In general, brain regions in the left hemisphere (particularly the hippocampus) appear to be more involved in encoding while the right hemisphere (particularly the prefrontal cortex) appears to be more engaged in the retrieval of episodic memory [22]. A specific set of regions, termed the “default mode network” (DMN) has been recently proposed to play a key role in memory, on the basis of rs-fMRI findings [23, 24]. The DMN includes the posterior cingulate, extending into the precuneus, lateral parietal, and medial prefrontal regions. This network has been shown to be more metabolically active at rest, decreasing its activity during challenging cognitive tasks. Interestingly, recent fMRI studies have suggested that the DMN needs to be disengaged or “deactivated” during successful memory formation [25, 26].

In patients with AD, a number of fMRI studies have reported decreased activation in the MTL region compared with older healthy subjects during episodic encoding tasks [27–29] (for a review, see Sperling and colleagues [8–10, 30]). A recent quantitative meta-analysis [31] demonstrated decreased activation of memory encoding-related regions (hippocampal formation, ventrolateral prefrontal cortex, precuneus, cingulate gyrus, and lingual gyrus) in AD patients. Other fMRI studies have also suggested that the DMN exhibits markedly abnormal responses during memory tasks in AD patients [32, 33]. Interestingly, the regions demonstrating aberrant DMN activity overlap anatomically with regions showing a high amyloid burden in early AD [34]. 

Regarding MCI patients, fMRI results have been markedly variable, ranging from findings of hyperactivation [27, 35] to hypoactivation [36, 37] of the MTL region. This variability in the results of previous studies is thought to be closely related to two factors; first, differences in subjects' ability to perform the fMRI task, and second, differences in the severity of the cognitive impairment along a continuum between normal aging and dementia [30]. MTL hyperactivation may be a compensatory response to maintain memory performance in the setting of early AD pathology [30]. As in AD, there is evidence that areas of the DMN are significantly affected in subjects with MCI relative to controls [32, 38]. A more recent study using a face-name memory task reported that a quantitative goodness-of-fit index of DMN connectivity was able to distinguish MCI patients who converted to AD from those who remained stable over a 2- to 3-year follow-up period [39].

### 2.2. Visuospatial Perception

Besides impairment of episodic memory, higher visual dysfunction is one of the cognitive hallmarks of AD [40]. Various visual functions, including the perception of objects, faces, words, and visuospatial stimuli are impaired in AD. However, deficits of visuospatial perception (i.e., the perception of space and motion) are the most prominent. Such deficits play a critical role in the navigational impairment found in AD and MCI [41–43]. 

**Figure 1:**
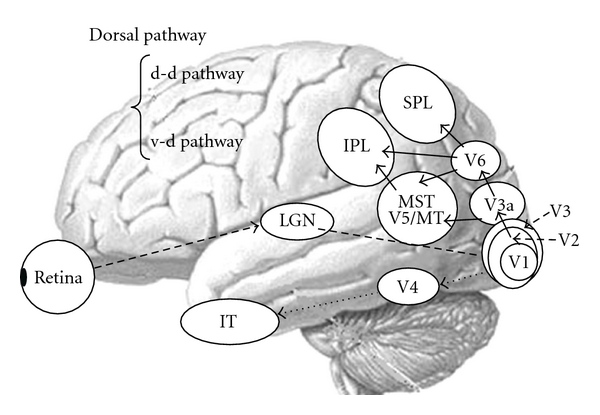
The parallel visual pathways in humans. Abbreviations in this and subsequent figures: d-d pathway, dorsodorsal pathway; v-d pathway, ventrodorsal pathway; LGN: lateral geniculate nucleus; V1, 2, 3, 4, and 6, primary, secondary, tertiary, quaternary, and senary visual cortices; V3a, V3 accessory; V5/MT: quinary visual cortex/middle temporal area; MST: medial superior temporal area; IPL, inferior parietal lobule, SPL: superior parietal lobule; IT: inferior temporal cortex.

Visual information is processed via parallel channels, namely, the parvocellular (P) and magnocellular (M) pathways [44] (for review, see Tobimatsu and Celesia [6]). Both systems begin in the retina and project to the primary visual cortex (V1) via the lateral geniculate nucleus. From V1, the P-pathway projects to the ventral stream, which includes V4 and the inferior temporal cortex. This system is responsible for processing form and color [6, 44]. Conversely, after V1, the M-pathway projects to the dorsal stream, which includes V3a, V5/MT+ (V5/MT and MST), V6, and the posterior parietal lobule. This system plays an important role in detecting space and motion information [6, 44]. Recently, one study reported that the dorsal stream is divided into two functional streams: the dorsodorsal (d-d) and ventrodorsal (v-d) streams [45]. The former consists of V6 and the superior parietal lobule (SPL) whereas the latter is formed by V5/MT and the inferior parietal lobule (IPL). Another series of studies demonstrated that macaque V6 is connected with visual areas including V1-3, V3a, V5/MT+, SPL, and IPL [46, 47]. A schematic diagram of the parallel visual pathways is shown in Figure 1. The distribution of neurofibrillary tangles and amyloid deposits in the visual system in typical AD has been described. Neurofibrillary tangles have been found in the visual association areas while amyloid plaques were reported to be uniformly distributed across the primary and association visual areas [4, 48–51]. In areas along the dorsal pathway, there is a significant loss of long corticocortical projections from early visual areas to V5 [52]. Thus, it is likely that the dorsal visual pathway is more susceptible to putative AD-related neuropathological changes than in the ventral pathway. 

Based on the findings mentioned above, several fMRI studies have been conducted to elucidate the neural basis of visuospatial impairment in AD patients [53–55]. Information regarding motion perception is independently described in the next section (Section 2.3). Thulborn et al. [53] demonstrated a reduction in right parietal activation while perceiving a visual saccade task. Similarly, Prvulovic et al. [54] reported reduced activation in the SPL, and compensatory recruitment of occipitotemporal cortex (ventral pathway) during the angle discrimination task. Bokde et al. [55] investigated the function of the parallel visual pathways in healthy controls and AD patients, finding that the control group exhibited selective activation of the ventral and dorsal pathways during face- and location-matching tasks. However, no such selective activation was observed in the AD group. Instead, the AD group recruited additional activation in the parietal and frontal lobes during the location-matching task. In contrast, there was no significant difference in activation between the two groups during the face-matching task. These fMRI results thus support the notion that the dorsal visual pathway is more susceptible to putative AD-related neuropathological changes than in the ventral pathway. 

In a study of MCI patients, Bokde et al. [57] measured activation changes in the parallel visual pathways using face matching and location-matching tasks. The healthy control group but not the MCI group exhibited selective activation of the ventral and dorsal pathways during the face- and location-matching tasks. During the face-matching task, there was no significant difference in activation between the two groups. However, both visual pathways were activated in the MCI group, possibly reflecting a compensatory mechanism, and increased activation was observed in the left frontal lobe during the location-matching task. Vannini et al. [58] investigated visuospatial processing in progressive and stable MCI patients during an angle discrimination task with varying task demands. Compared with stable MCI patients and controls, progressive MCI patients exhibited a stronger relationship between task demand and brain activity in the left SPL. The authors concluded that increased parietal activation in progressive MCI patients could reflect a reduction in neuronal efficacy in the dorsal pathway due to accumulating AD pathology.

### 2.3. Motion Perception

To our knowledge, there have been two fMRI studies investigating motion perception in AD (but not MCI) patients [59, 60]. Thiyagesh et al. [59] used depth and motion stimuli, reporting that an AD group exhibited hypoactivation in V5, SPL, parietooccipital cortex, and the premotor cortices, as well as greater compensatory activation in IPL. Thiyagesh et al. [60] also examined the treatment effects of acetylcholinesterase inhibitors in AD patients, recording fMRI while subjects performed motion perception tasks. They reported increased activation in the left precuneus, left cuneus, left supramarginal gyrus, right parieto-temporal cortex and right IPL after treatment. Furthermore, increased activation in the left precuneus was found to correlate significantly with improved functioning in terms of activities of daily living. These findings suggest that fMRI scanning during motion perception tasks could be useful for monitoring the efficacy of disease modifying therapies. However, further fMRI studies are necessary to elucidate the neural basis of impaired motion perception in patients with AD and MCI. 

Motion information is mainly processed by the dorsal stream including V3a, V5/MT+ (V5/MT and MST), V6, and the posterior parietal lobule [6, 44–47, 61]. In particular, the lateral motion area V5/MT+ (V5/MT and MST) and medial motion area V6 have been recently considered key structures in extrastriate motion processing [61]. Human V5/MT+ is functionally and anatomically located in the depths of the anterior occipital sulcus (the ascending limb of the inferior temporal sulcus) and the anterior portions of either the inferior lateral occipital or the inferior occipital sulcus. V5/MT has a high density of motion-sensitive neurons. V1 neurons have small receptive fields that can detect local motion. The properties of V5/MT neurons include larger receptive fields than V1, center-surround interactions, integration of different directions, and sensitivity to motion coherence. Taken together, these findings suggest that V5/MT integrates local motion signals from V1 into the more global representations of motion needed as a basis for perceptual performance. Projections from V5/MT into the neighboring MST area and into the parietal lobe appear to provide a good neural substrate for the use of visual motion in the control of eye movements and other actions (for review, see Tobimatsu and Celesia [6]; Yamasaki et al. [56]). 

Recent findings have led to V6 being considered an additional medial motion area [61], located in the parieto-occipital sulcus of macaques and humans. In macaques, V6 abuts the end (the representation of the far periphery) of V3 and V3a. It has a clear retinotopic organization, representing the contralateral hemifield. Most of its cells are visually responsive, and approximately 75% are direction sensitive. Similar to V5/MT+ cells, the receptive field of cells in V6 is much larger than that of cells in V1. Adjacent area V6a, which occupies the dorsal/anterior portion of the sulcus, has no obvious retinotopic organization and only around 60% of the neurons are visually responsive. Visual neurons are again predominantly motion sensitive. These findings suggest that macaque V6 and V6a play a pivotal role in providing visual motion information to the motor system. Similarly, human V6 is confined to the dorsal portion of the parieto-occipital sulcus, occupying the fundus and posterior bank of the sulcus. This area contains a complete representation of the contralateral hemifield, with the lower field located medially and more anterior to V3/V3a, extending dorsally to the upper field. As in primates, human V6 responds to coherent more than incoherent motion (for review, see Fattori et al. [46]). 

**Figure 2:**
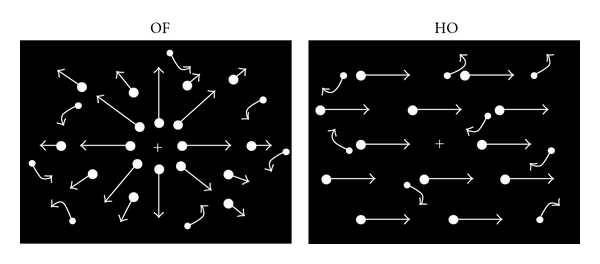
Visual motion stimuli. Four hundred white square dots (visual angle, 0.2 × 0.2°; luminance, 48 cd/m^2^) were randomly presented on a black background (visual angle, 50 × 48°; luminance, 0.1 cd/m^2^). The contrast level was 99.6%. The white dots moved at a velocity of 5.0°/s. When the white dots move incoherently, random motion stimulation is created. When the white dots move coherently, OF and HO are perceived. Abbreviations in this and subsequent figures: HO: horizontal motion; OF: optic flow (adapted from Yamasaki et al., in press [56]).

Many types of motion stimuli have been applied to examine motion processing in healthy humans and several neurological disorders. In particular, coherent motion stimuli using random dots have been widely used in psychophysical, electrophysiological, and neuroimaging studies to investigate global motion processing [62–64]. There are several types of global motion, including radial optic flow (OF) and horizontal motion (HO; Figure 2). Radial OF, the visual motion perceived during observer self-movement, is particularly important for daily life because it provides cues about the direction and three-dimensional structure of the visual environment [65, 66] (for review, see Tobimatsu and Celesia [6]; Yamasaki et al. [56]). In primates, MST and posterior parietal neurons have been found to selectively respond to OF [67, 68] while V5/MT neurons do not appear to exhibit such specific selectivity [69]. Several neuropsychological studies have reported that patients with AD exhibited impaired radial OF perception, associated with visuospatial disorientation, but preserved HO perception [70, 71]. Some patients with MCI have also been found to exhibit selective impairments in OF perception [43]. These findings suggest differential processing of OF and HO. fMRI studies with healthy subjects have demonstrated that several areas within the dorsal streams are activated by OF [64, 72–74]. However, it remains unknown how OF and HO are differentially processed within the two distinct dorsal streams (the v-d and d-d streams) in healthy subjects as well as in patients with cognitive decline.

### 2.4. Brain Connectivity in the Resting State

As mentioned above, a number of fMRI studies have revealed that patterns of activation or deactivation during task performance are altered in AD and MCI patients. However, these task-based imaging paradigms require the active participation of subjects, which may be difficult for some AD and MCI patients, depending on the task demands involved. This problem can be resolved by the development of rs-fMRI, a technique that has attracted substantial research attention. rs-fMRI signals are thought to reflect spontaneous neuronal activity and/or the endogenous/background neurophysiological processes of the brain in the resting state [75–77]. Because no stimulation or task-related responses are required, this method has practical advantages for clinical applications and can be used easily even for patients with severe dementia [14]. Various methods exist for analyzing rs-fMRI data: seed region of interest (ROI) based on functional connectivity analysis, independent components analysis, clustering, pattern classification, graph theoretical analysis, and “local” methods, including the measurement of regional homogeneity. The characteristics of each of these methods have been described in detail elsewhere (for a review, see Margulies et al. [78]). 

Spontaneous brain activity is thought to be organized by synchronized oscillations at different temporal and spatial scales [79]. Temporal correlations between low-frequency oscillations of fMRI signals derived from distinct brain areas at rest reflect the spatial aspects of this organization. Biswal et al. [75] reported the first such findings of at-rest functional connectivity in the somatomotor system in healthy subjects. Since then, rs-networks have been reported for many functional systems, including the motor, primary sensory, language, attention, and DMN systems [80–84]. In the past several years, many researchers have begun to study the pathophysiology of AD and MCI by investigating changes in rs-fMRI signals (for a review see Liu et al. [14], Sorg et al. [15], and Filippi and Agosta [16]). An rs-fMRI study using seed ROI analysis demonstrated disrupted left-right hippocampal connectivity in AD patients [85]. Other seed-ROI rs-fMRI studies of AD patients reported altered connectivity between the hippocampus and several neocortical regions including the posterior cingulate cortex (PCC), lateral temporal cortex, medial, and lateral prefrontal cortices, and inferior parietal cortex [86–88]. A study of MCI patients revealed decreased functional connectivity between the PCC and temporal cortex compared to controls [89]. Whole-brain analyses of MCI patients have also reported diffuse alterations of connectivity in the DMN compared with healthy elderly controls [90, 91]. For example, Sorg et al. [90] revealed decreased DMN connectivity not only in the PCC and the bilateral parietal cortex, but also in the right medial prefrontal cortex. Qi et al. [91] reported decreased functional activity in regions associated with the DMN, including the bilateral precuneus/PCC, right IPL and left fusiform gyrus, and a trend towards decreased right medial temporal lobe activity. In contrast, enhanced rs-functional connectivity in frontal regions has been reported in AD patients [92–94]. Increased functional connectivity between regions of the DMN and frontal areas has also been reported in AD [93] and MCI patients [89, 91]. These findings suggest that patients with AD may rely on increased prefrontal connectivity to compensate for reduced temporal lobe function. 

Disruption of global functional organization has also been reported in AD [92, 94]. Wang et al. [94] found that AD patients exhibited decreased positive correlations of activity between the prefrontal and parietal lobes, but increased positive correlations within the prefrontal, parietal, and occipital lobes. These findings provide further evidence for the notion that anterior-posterior disconnection and increased within-lobe functional connectivity occur in AD. Supekar et al. [92] investigated small-world properties in the brain, namely, the clustering coefficients and characteristic path lengths of 90 brain nodes in AD patients, as indices of global functional organization. Clustering coefficients are considered a measure of local network connectivity, and networks with high average clustering coefficients are characterized by densely connected local clusters. The characteristic path length is a measure of how well connected a network is. A network with a low characteristic path length exhibits short distances between any two nodes. Small-world networks are characterized by a high clustering coefficient and a low characteristic path length. Based on graph theoretical analysis, AD patients were found to exhibit a loss of small-world properties in the brain, with a significant reduction in the clustering coefficient. This finding suggests the involvement of disrupted local connectivity in the disease [92]. In addition, a trend towards randomness in brain networks in AD was recently reported by Sanz-Arigita et al. [95] using graph analysis. A post hoc analysis of regional synchronization revealed increased synchronization in AD involving the frontal cortices and occipital regions. This translates into a global reduction of functional long-distance links between frontal and caudal brain regions. 

Recently, Chen et al. [96] succeeded in classifying patients as AD, MCI, and cognitively normal subjects using a large-scale network analysis. The altered connectivity patterns among the cortical networks were significantly correlated with the results of cognitive tests. Zhang et al. [97] investigated alterations in PCC functional connectivity by comparing a healthy control group with three separate AD groups (mild, moderate, and severe AD) using a method of temporal correlation. They found that modulation of the DMN with abnormal PCC connectivity was able to change along with AD stage progression. Thus, changes in functional connectivity of the resting brain may provide an imaging marker for monitoring AD progression.

## 3. fMRI with Additional ERP Recording in Motion Perception

fMRI is characterized by excellent spatial resolution, but low temporal resolution. Thus, it can only discriminate between events that are separated by several seconds. In contrast, the temporal resolution of ERPs is in the order of milliseconds, which is far superior to that of other neuroimaging methods including fMRI. However, ERPs offer poor spatial resolution compared with fMRI. Based on these characteristics, the combined use of fMRI and ERPs is considered to be an extremely useful technique for evaluating the spatiotemporal functional changes in AD and MCI in detail. In the following section, we summarize the findings of our studies using fMRI and separate ERP measurement during the performance of motion perception tasks in healthy controls and MCI patients [56, 98–102]. 

### 3.1. Two Distinct Motion Pathways

**Figure 3:**
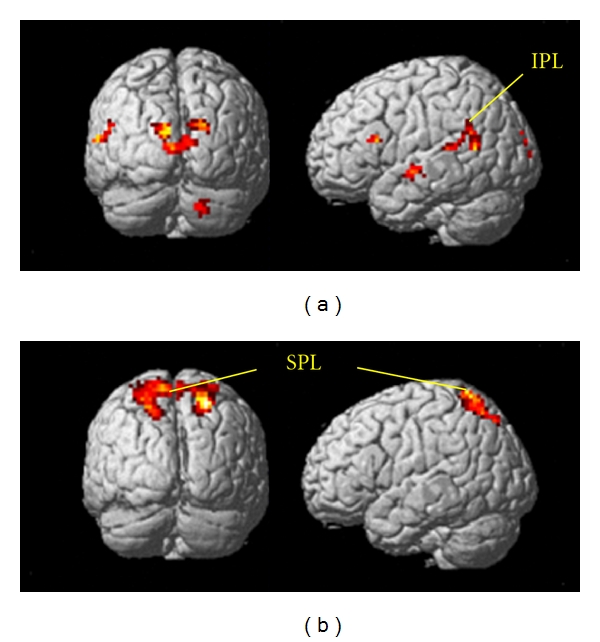
fMRI responses in healthy young adults. (a) Activations of the v-d pathway including the IPL (BA 39/40) are found in the OF minus HO condition. (b) The HO minus OF condition shows the activation of the d-d pathway, including the SPL (BA 7; adapted from Yamasaki et al., in press [56]).

As stated in Section 2.3, it is currently unclear how coherent OF and HO are differently processed in the v-d and d-d streams in humans. We first examined the neural basis of motion perception in healthy young adults by measuring fMRI and visual ERPs during the perception of coherent OF and HO stimuli [56, 98–102]. In our fMRI experiments, we used a block design rather than an event-related design to detect subtle differences in BOLD signals between the responses to the two types of motion stimuli. To complement temporal information associated with neural activity, high-density 128-channel ERPs were separately recorded. Our visual stimuli consisted of 400 white square dots randomly presented on a black background. The white dots moved at a velocity of 5.0 degrees of visual angle per second. The dots in the HO stimuli moved leftward or rightward while those in the OF stimuli moved radially in an outward pattern (Figure 2). The coherence level was 90% for both types of stimuli. We used random motion (RM) as a baseline in our analysis to suppress the neural activity of nondirectional neurons. When fMRI was recorded during OF and HO perception, the OF stimulus significantly activated the v-d stream, including the IPL (BA 39/40) in the OF minus RM-baseline and OF minus HO contrasts (Figure 3(a)). There was no significant activation in other motion areas, such as V3a, V5/MT+, V6, and the d-d stream (SPL) in either contrast. On the contrary, the d-d stream, including the SPL (BA 7) was significantly activated during perception of the HO stimulus in the HO minus RM-baseline and the HO minus OF contrasts (Figure 3(b)). However, significant activations of V3a, V5/MT+, and the v-d stream (IPL) were not observed in either contrast. These findings indicate that OF and HO motions are processed differently within the dorsal stream. The d-d stream (SPL) appears to be more closely related to HO motion processing while the v-d stream (IPL) is important for OF motion processing [56, 98, 102]. In addition, the lack of activation in V3a, V5/MT+ and V6 suggests that these brain regions do not distinguish between RM (or incoherent), coherent OF, and HO motion. Previous studies have reported that the v-d stream, including the IPL, plays crucial roles in high-level motion perception, space perception, and action organization [45, 103, 104]. Conversely, the SPL is highly responsive to unidirectional coherent motion stimuli [105]. Therefore, the IPL appears to play an important role in complex OF processing while activity in the SPL is related to the processing of simple unidirectional motion. 

**Figure 4:**
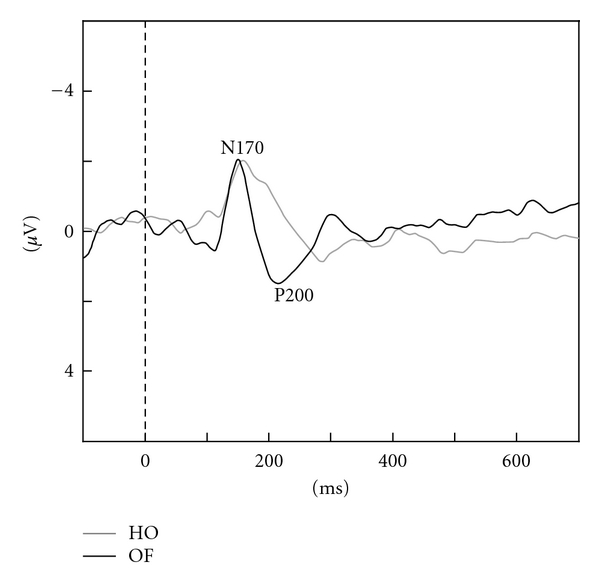
ERP responses in healthy young adults. Two major components (N170, P200) were observed. The N170 was evoked by both HO and OF stimuli while the P200 was only elicited by OF stimuli (adapted from Yamasaki et al., in press [56]).

Using the same motion stimuli, we conducted several experiments using high-density 128-channel ERP recording. The results revealed that perception of these stimuli was associated with two major ERP components (N170, P200; Figure 4). The occipitotemporal N170 had a V5/MT origin, and was evoked by both types of stimuli [56, 98]. In contrast, the parietal P200 originated in IPL (BA 40) and was only elicited by OF stimuli [56, 98]. These findings indicate that the N170 component is a nonspecific, motion-related component originating from V5/MT, while the P200 is an OF-specific component generated by the IPL [56, 98]. Thus, our ERP studies provided further evidence suggesting a close relationship between the v-d stream (IPL) and OF perception. On the basis of these findings, we conclude that different types of spatiotemporal processing are driven by OF and HO motion stimuli within two distinct dorsal streams in healthy young adults.

### 3.2. Effects of Aging and Cognitive Decline on Motion Perception

Based on our previous fMRI and ERP findings in healthy young adults [56, 98], we conducted a preliminary investigation of motion perception in healthy older elderly adults and MCI patients. In fMRI, healthy elderly adults showed similar activation patterns to healthy young adults. That is, OF stimulus perception dominantly activated the v-d stream (IPL) while the d-d stream (SPL) was more strongly activated by HO stimuli. Interestingly, in MCI patients, IPL activation in response to OF stimuli was decreased compared to healthy elderly adults. In contrast, there was no apparent difference in SPL activation for HO between healthy old adults and MCI patients. These results imply that the function of the v-d stream (IPL) is selectively impaired in MCI while d-d (SPL) function appears to be preserved. 

Two major ERP components (N170, P200) were detected during this task in both healthy old adults and MCI patients, as observed in healthy young adults [98]. MCI patients exhibited prolonged OF-specific P200 latencies compared to healthy elderly adults, but no differences were found in N170 latency. This finding suggests that the function of the v-d stream (IPL) related to OF perception is selectively impaired in MCI patients, consistent with fMRI findings. These results are in accord with the functional importance of the IPL in MCI patients [106]. Taken together, our findings provide evidence that fMRI with additional ERP recording can be useful for detecting spatiotemporal functional changes in the brain, including the IPL, in MCI patients.

## 4. Conclusion

The most prominent symptoms of MCI and AD are memory loss and visuospatial impairments. The task-related fMRI studies discussed in this review demonstrated specific alterations of several brain functions, such as memory networks and visuospatial perception. Decreased activation in the distributed networks including the MTL and DMN has been observed during the encoding of new memories. Alteration of memory networks can distinguish AD converters of MCI from nonconverters. During visuospatial perception, the activation of the dorsal pathway was reduced in conjunction with compensatory activation in the ventral pathway and frontal regions. Repeated fMRI scanning during a visuospatial task revealed enhanced activation in the dorsal stream after treatment. This activation was correlated with improvements on neuropsychological assessments. Studies utilizing the recent development of rs-fMRI have also demonstrated altered functional connectivity within the hippocampus, DMN, and larger-scale networks. Changes in the functional connectivity of the resting brain have been successfully used in the classification of patients with AD, MCI, and the healthy elderly. Taken together, the studies reviewed above indicate that fMRI is useful as an early diagnostic aid for AD and MCI patients. Furthermore, fMRI is a potentially useful method for monitoring the progression of disease and the efficacy of disease-modifying therapies. Unfortunately, the temporal resolution of fMRI is far inferior to that of ERPs. By recording both fMRI and ERP activity in response to the same motion stimuli, we revealed spatiotemporal functional changes of the IPL in MCI patients during coherent motion perception. Therefore, the use of ERPs appears to provide additional information about spatiotemporal functional changes in MCI.
